# Abscisic Acid Standardized Fig (*Ficus carica*) Extracts Ameliorate Postprandial Glycemic and Insulinemic Responses in Healthy Adults

**DOI:** 10.3390/nu11081757

**Published:** 2019-07-31

**Authors:** Fiona S. Atkinson, Agusti Villar, Anna Mulà, Andrea Zangara, Ester Risco, Carsten R. Smidt, Raquel Hontecillas, Andrew Leber, Josep Bassaganya-Riera

**Affiliations:** 1 School of Life and Environmental Sciences and Charles Perkins Centre, D17, The University of Sydney, Sydney, NSW 2006, Australia; 2 Euromed S.A., C/ Rec de Dalt, 21-23, Pol. Ind. Can Magarola, 08100 Mollet del Valles, Barcelona, Spain; 3 Centre for Human Psychopharmacology, Swinburne University, Melbourne, VIC 3122, Australia; 4 Smidt Labs, LLC, Sandy, UT 84092, USA; 5 BioTherapeutics, Inc, 1800 Kraft Drive, Suite 200, Blacksburg, VA 24060, USA

**Keywords:** abscisic acid, fig fruit extract, glycemic index, insulinemic index

## Abstract

Abscisic acid (ABA) can improve glucose homeostasis and reduce inflammation in mammals by activating lanthionine synthetase C-like 2 (LANCL2). This study examined the effects of two fig fruit extracts (FFEs), each administered at two different ABA doses, on glycemic index (GI) and insulinemic index (II) to a standard glucose drink. In a randomized, double-blind crossover study, 10 healthy adults consumed 4 test beverages containing FFE with postprandial glucose and insulin assessed at regular intervals over 2 h to determine GI and II responses. Test beverages containing 200 mg FFE-50× and 1200 mg FFE-10× significantly reduced GI values by −25% (*P* = 0.001) and −24% (*P* = 0.002), respectively. Two lower doses of FFE also reduced GI values compared with the reference drink (by approximately −14%), but the differences did not reach statistical significance. Addition of FFE to the glucose solution significantly reduced II values at all dosages and displayed a clear dose-response reduction: FFE-50× at 100 mg and 200 mg (−14% (*P* < 0.05) and −24% (*P* = 0.01), respectively) and FFE-10× at 600 mg and 1200 mg (−16% (*P* < 0.05) and −24% (*P* = 0.01), respectively). FFE supplementation is a promising nutritional intervention for the management of acute postprandial glucose and insulin homeostasis, and it is a possible adjunctive treatment for glycemic management of chronic metabolic disorders such as prediabetes and type 2 diabetes mellitus.

## 1. Introduction

Impaired glucose tolerance (IGT), or prediabetes, an intermediate state with plasma glucose levels ranging between normoglycemia and diabetes, is an important health concern [1,2]. In 2017, approximately 352 million people worldwide were living with IGT, and its prevalence is projected to increase to 587 million by 2045 [2]. The Centers for Disease Control and Prevention estimated that 84 million adults in the US had prediabetes in 2015, which represented approximately 34% of the adult population at the time [3]. Metabolic syndrome, an independent risk factor for both microvascular and macrovascular diseases, is closely related to IGT and insulin resistance. In 2017, approximately USD 44 billion was spent on healthcare due to prediabetes in the US [2]. Moreover, people with IGT and prediabetes are at a high risk of progressing to type 2 diabetes mellitus [1], potentially creating an even larger burden on the health system.

Prediabetes management involves dietary and lifestyle modifications, along with weight loss in obese individuals, with a primary goal of restoring normoglycemia and maintaining β-cell function [4,5]. Plant-based supplements are generally safer with fewer side effects compared with synthetic drugs [6,7] and, therefore, may offer promising options for the prevention and treatment of chronic diseases, such as type 2 diabetes mellitus. Abscisic acid (ABA), a phytohormone commonly present in fruits and vegetables, has been shown in mammals to not only promote peripheral glucose uptake [8,9] but also to possess adaptogenic properties related to the stress response [10]. Long-term oral administration of exogenous ABA reduced fasting plasma glucose concentrations and ameliorated glucose tolerance in leptin receptor-deficient (db/db) mice [9]. Mechanistically, the ABA receptor has been proposed to be lanthionine synthetase C-like 2 (LANCL2) [11]. The signaling pathway downstream of LANCL2 includes a G-protein-mediated activation of adenylate cyclase, cAMP production, and activation of protein kinase A [12]. In addition, LANCL2 can facilitate phosphorylation of Akt by mTORC2 via direct physical interactions [13]. Active mTORC2 causes translocation of GLUT4 to the plasma membrane and stimulates glucose uptake [14]. 

In healthy adults, plasma concentration of ABA increases following oral glucose administration, stimulating peripheral glucose uptake [15]. Individuals with diabetes have been shown to have suboptimal levels of endogenous ABA [16,17]. Moreover, at nanomolar concentrations, ABA stimulates glucose uptake in a manner similar to insulin [15]. Taken together, these studies suggest that ABA may play an important role in modulating glucose homeostasis in humans [18]. In fact, intake of an aqueous fruit extract containing ABA at <1 µg/kg of body weight lowered glycemia and insulinemia during a standard meal in healthy volunteers [19]. ABA has received self-affirmed generally recognized as safe (GRAS, US FDA) status, and it has been evaluated as a safe substance by the US Environmental Protection Agency (EPA), posing no dietary risks to humans. Figs (*Ficus carica* L., *Moraceae*) are an important source of ABA (2812 pmol/g wet weight) [19]. Fig fruit extracts (FFEs) may potentially provide a natural method for controlling blood glucose and insulin fluctuations in metabolic and nutritional disorders, such as hyperglycemia and insulin resistance. 

Glycemic index (GI) and insulinemic index (II) are common parameters used to assess the glycemic impact of foods, beverages, and dietary supplements. GI is a measure of carbohydrate quality and ranks products according to the extent to which the available carbohydrates in foods and beverages raise blood glucose compared to an equal carbohydrate portion of glucose [20]. The aim of this study was to determine the effects of two FFEs, standardized in ABA content and administered at two different dose levels, on postprandial glycemic and insulinemic responses relative to a standard glucose drink in healthy adults.

## 2. Materials and Methods 

### 2.1. Study Design and Participants

This was a single-center, randomized, double-blind crossover study. The study was conducted using internationally recognized methods validated in multicenter research trials [21,22]. All experimental procedures were performed in accordance with international standards for conducting ethical research with humans and were approved by the Human Research Ethics Committee of the University of Sydney (Protocol: 2013/766). All participants provided written, informed consent before participation.

Ten healthy adults aged between 18–45 years, with a body mass index (BMI) between 18–25 kg/m^2^ and normal glucose tolerance, were recruited from a participant database from the Sydney University Glycemic Index Research Service. Normal glucose tolerance was assessed using the results from an oral glucose tolerance test conducted within the previous 1 month prior to participation in this study (fasting glucose <5.5 mmol/L and 2 h postprandial glucose <7.8 mmol/L). Participants were excluded if they were over- or underweight, were dieting, had IGT, any illness, or food allergies, or were regularly taking any prescription medication other than standard contraceptives.

Participants completed 7 test sessions each on a different day, with consecutive sessions separated by at least 1 day. Each participant tested the reference food (oral glucose solution containing 50 g of available carbohydrate) on sessions 1, 4, and 7 and one of the four test beverages during each of the remaining sessions in a random, counterbalanced order. Participants consumed the reference glucose drink on three separate occasions and each test beverage on one occasion only. Participants maintained their usual dietary and lifestyle patterns throughout the study.

### 2.2. Study Treatments

Pharmaceutical-grade dried FFEs (ABALife^TM^, Euromed, Spain) were produced from *Ficus carica* L. fruit using a sophisticated, patent-pending process (EP17382616.5) and were standardized in ABA content. ABA content in these extracts was determined using reversed-phase ultra-high-performance liquid chromatography (UHPLC). Fig extracts contained one of two ABA concentrations: FFE-10X (ABA ≥300 ppm by UHPLC, drug extract ratio (DER) of native extract is 50–60:1) and FFE-50× (ABA ≥50 ppm by UHPLC, DER of native extract is 7–1:1). Two different concentrations of ABA (10× or 50×) were used in this study to ensure that any effects on glycemic and insulinemic responses were due to ABA content and not to other compounds in the fig matrix. Each extract (FFE-10× or FFE-50×) was tested at two doses equivalent to 40 µg (lower dose) and 80 µg (higher dose) ABA. 

The reference beverage and the four test beverages all contained 50 g of available carbohydrate in the form of an oral glucose solution prepared as 51.4 g Glucodin™ powder (Valeant Pharmaceuticals, Australia) dissolved in 250 mL water. The oral glucose solutions for the reference and test beverages were prepared the day before required and stored in the refrigerator overnight. The FFE required for each test beverage was added into the glucose solution immediately prior to being served to a participant. The four FFE treatments were 100 mg FFE-50×, 200 mg FFE-50×, 600 mg FFE-10×, and 1200 mg FFE-10×. The nutritional contents of the reference glucose drink and test beverages were identical, except for addition of the FFE powder in the test beverages. Both participants and researchers were blinded to the differences between the extracts and the ABA doses of the test beverages throughout the study.

### 2.3. Study Procedures

The night before each test session, participants consumed a carbohydrate-based evening meal, excluding legumes and alcohol, and then arrived at the research center in the morning after fasting overnight for at least 10 h. Two fasting capillary blood samples were collected from a warmed hand (≥0.5 mL blood into 1.5 mL tubes containing 10 IU heparin) from each participant before administration of the reference or test beverage. The 2 fasting samples were collected 5 min apart, and the average concentration of these timepoints was used as the baseline concentration. The reference glucose drink or test beverage (containing 100 or 200 mg FFE-50× or 600 or 1200 mg FFE-10×; both extract doses equivalent to either 40 or 80 µg ABA, respectively, mixed in the reference glucose drink) was administered along with 250 mL of plain water, which was consumed within 12 min. Additional finger-prick blood samples were collected at 15, 30, 45, 60, 90, and 120 min after starting consumption of the reference or test beverage. 

Capillary blood samples were centrifuged at 10,000× *g* for 45 s immediately after collection. Plasma samples were then immediately transferred into labeled, uncoated tubes and stored at −30 °C for later analysis. Plasma glucose concentrations were evaluated in duplicate using a glucose hexokinase enzymatic assay (Beckman Coulter Inc., Brea, CA, USA.) on an automatic centrifugal spectrophotometric clinical chemistry analyzer (Beckman Coulter AU480^®^, Beckman Instruments Inc., Brea, CA, USA). Plasma insulin concentrations were measured using an insulin sandwich type enzyme-linked immunoassay (Insulin ELISA kit, ALPCO^®^, Salem, NH, USA), respectively. All seven test sessions for each participant were analyzed within the same assay.

### 2.4. Data Analysis and Statistical Analysis 

The incremental area under the plasma glucose or insulin response curves over 2 h (iAUC), ignoring the area below fasting concentration, were calculated using the trapezoidal rule. Glycemic index (GI) and insulinemic index (II) values were then calculated for each participant’s test beverages by expressing the iAUC response for a given test beverage as a percentage of the average response produced by the reference beverage in the same individual [20]. Sample-size calculations based on data from published GI studies suggested 10 participants were required in order to detect a significant difference among GI and II values with 90% power [20]. Comparative analyses were performed on glucose and insulin variables (GI, II, iAUC and peak postprandial responses) using analysis of variance (ANOVA) and the least significant difference (LSD) test for multiple comparisons. IBM^®^ SSPS^®^ Statistic software 24 was used for all statistical analyses. Linear regression analysis was used to assess the association between ABA dose and postprandial GI or II responses of the beverages. No outlier responses were excluded from the analyses. Results are expressed as means with standard error of mean (SEM). Statistical significance was set at *P* < 0.05.

## 3. Results

### 3.1. Participant Characteristics

A total of 10 healthy adults (7 women and 3 men; 7 White/Caucasian, 1 Hispanic, and 2 Asian) with a mean (± standard deviation) age of 29.5 ± 10.1 years and an average body mass index of 22.1 ± 2.0 kg/m^2^ completed the study. All participants completed the 7 test visits and associated protocols (dropout rate: 0%). The mean within-individual coefficient of variation for the glycemic responses for the three repeated reference glucose solution treatments was 11%, which was within the accepted level of ≤30% [22]. 

### 3.2. Tolerability of Test Beverages

The FFE when consumed at both the lower doses (100 mg FFE-50× and 600 mg FFE-10×; equivalent to 40 µg ABA) and higher doses (200 mg FFE-50× and 1200 mg FFE-10×; equal to 80 µg of ABA) were palatable and well tolerated. No adverse events were reported.

### 3.3. Two-Hour Plasma Glucose and Insulin Responses for Reference Beverage and Test Beverages

**Figure 1:**
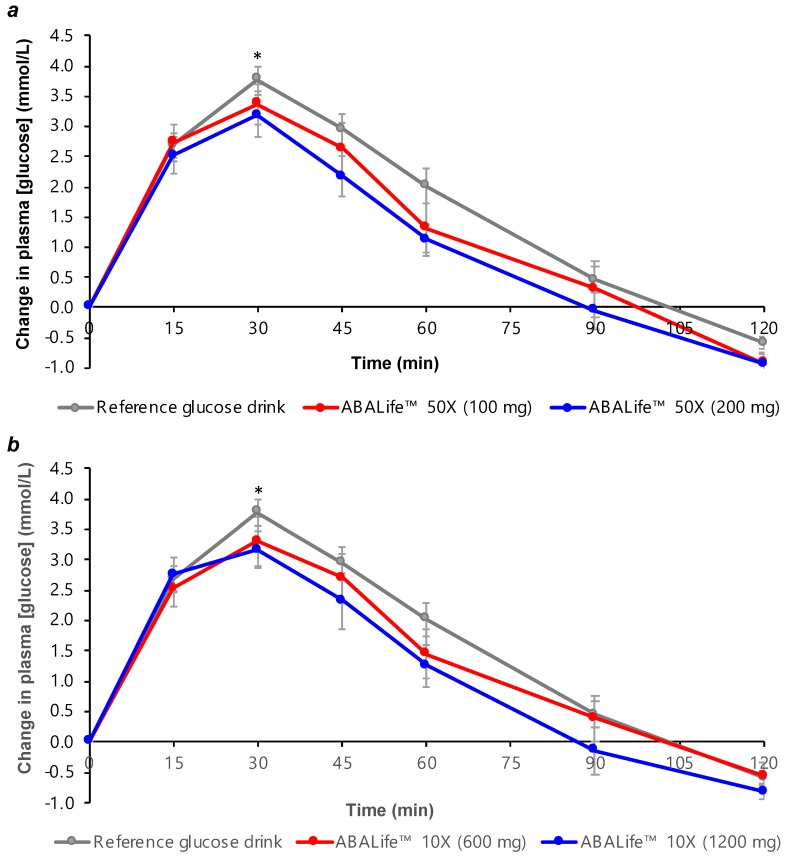
Change in postprandial 2 h plasma glucose concentration in healthy adults for (**a**) the two doses of abscisic acid (ABA) Life™ 50× fig fruit extract consumed with a glucose solution; (**b**) the two doses of ABA Life™ 10× fig fruit extract consumed with a glucose solution. Data are mean ± standard error mean (SEM), *n* = 10 for the 3 repeated tests in each participant for the reference glucose drink, and *n* = 10 for each test drink containing fig fruit extract (FFE). The two lower doses of each fig fruit extract contained 40 µg of abscisic acid (shown in red), and the two higher doses of each fig fruit extract contained 80 µg of abscisic acid (shown in blue). * indicates a significant difference between peak 30 min glucose concentration for the high ABA dose test drinks (200 mg FFE-50× and 1200 mg FFE-10×) compared to the reference food (*P* ≤ 0.037).

Following ingestion of the reference beverage or test beverage, average plasma glucose concentrations reached a peak at 30 min and gradually declined to preprandial levels by 120 min (Figure 1). Higher doses of the two FFEs (200 mg FFE-50× and 1200 mg FFE-10×) resulted in lower peak plasma glucose concentrations at 30 min compared to the reference glucose drink (*P* = 0.003 and *P* = 0.037, respectively). No significant differences were detected between the peak 30 min glucose response for the reference glucose drink and either of the lower FFE doses (100 mg FFE-50× and 600 mg FFE-10×). Although a trend towards lower peak 30 min glucose concentrations was observed for the higher FFE doses compared to the corresponding smaller doses of the same extracts, these differences did not reach significance. 

**Figure 2:**
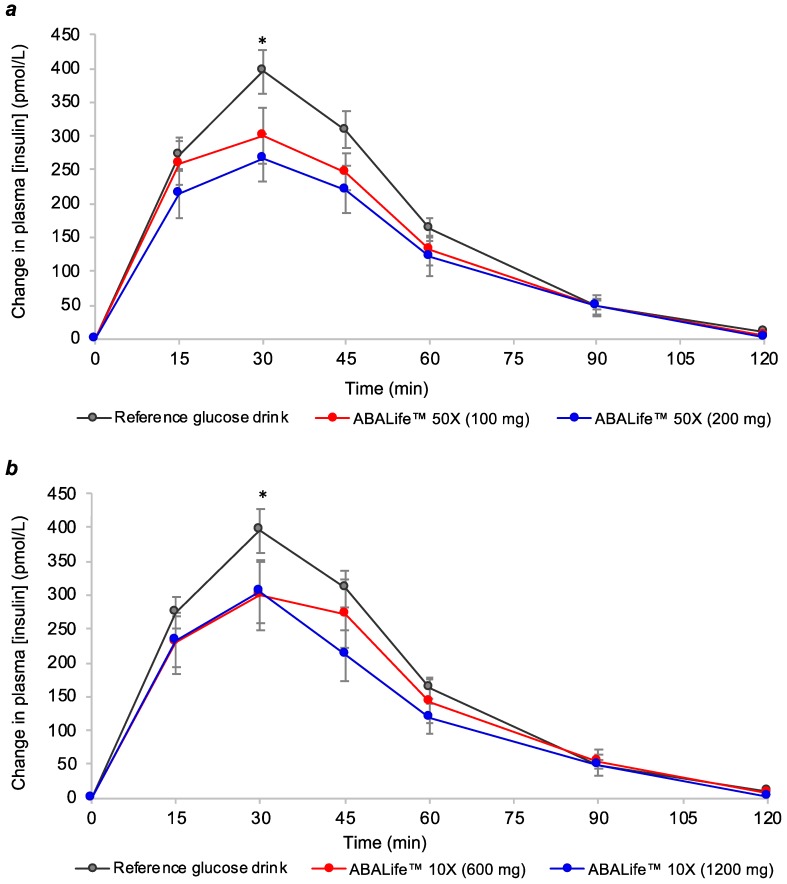
Change in postprandial 2 h plasma insulin concentration in healthy adults for (**a**) the two doses of ABA Life™ 50× fig fruit extract consumed with a glucose solution; (**b**) the two doses of ABA Life™ 10× fig fruit extract consumed with a glucose solution. Data are mean ± standard error mean (SEM), *n* = 10 for the 3 repeated tests in each participant for the reference glucose drink, and *n* = 10 for each test drink containing fig fruit extract (FFE). The two lower doses of each fig fruit extract contained 40 µg of abscisic acid (shown in red), and the two higher doses of each fig fruit extract contained 80 µg of abscisic acid (shown in blue). * indicates a significant difference between peak 30 min insulin concentration for each test drink containing FFE compared to the reference food (*P* ≤ 0.019).

Similar to plasma glucose response curves, the postprandial insulin response curves produced by the reference glucose solution and the four test beverages peaked at 30 min and gradually declined to preprandial levels by 120 min (Figure 2). All four FFE test beverages produced significantly lower peak plasma insulin concentrations at 30 min compared to the reference glucose drink (*P* ≤ 0.008). 

**Figure 3:**
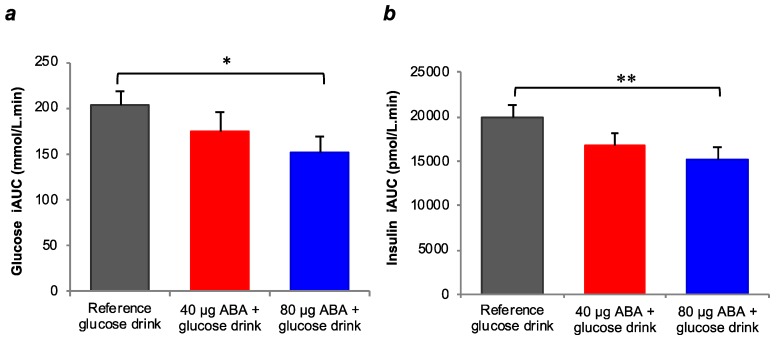
Two-hour incremental area under the curve (iAUC) responses for the two pooled lower 40 µg abscisic acid (ABA) doses (100 mg FFE-50× and 600 mg FFE-10×, *n* = 20, shown in red) and the two pooled higher doses of ABA (200 mg FFE-50× and 1200 mg FFE-10× *n* = 20, shown in blue) compared to the reference glucose drink (*n* = 30). (**a**) Postprandial glucose responses and (**b**) postprandial insulin responses. Data are shown as mean ± standard error mean (SEM). * indicates a significant difference between pooled higher ABA dose compared to the reference glucose drink at *P* = 0.042. ** indicates a significant difference between pooled higher ABA dose compared to the reference glucose drink at *P* = 0.021.

When data for the two test beverages containing 80 µg ABA (200 mg FFE-50× and 1200 mg FFE-10×) were combined together, the postprandial glycemic response was significantly lower than for the average 2 h glucose iAUC produced by the reference glucose drink (152 mmol/L·min vs 203 mmol/L·min, *P* = 0.042) (Figure 3). The two lower ABA dose test beverages (100 mg FFE-50× and 600 mg FFE-10×) produced a nonsignificant reduction in glucose iAUC compared to the reference beverage. When the two test beverages containing 80 µg of ABA were combined together (200 mg FFE-50× and 1200 mg FFE-10×), the postprandial insulinemic response was significantly lower than the reference glucose drink (15157 pmol/L·min vs 19983 pmol/L·min, *P* = 0.021) (Figure 3). The difference between the combined postprandial insulin iAUC for the two lower ABA dose beverages (100 mg FFE-50× and 600 mg FFE-10×) and the reference drink did not reach statistical significance. 

### 3.4. Glycemic Index and Insulinemic Index of Test Beverages

The addition of FFE to a glucose solution produced considerable reductions in both GI and II compared to the glucose solution consumed alone (Table 1). The two test beverages containing the higher dose of 80 µg ABA (200 mg FFE-50× and 1200 mg FFE-10×) significantly lowered GI by –25% (*P* = 0.001) and –24% (*P* = 0.002), respectively, and II by –24% (both *P* = 0.001) compared to the reference glucose solution. Similarly, the two lower 40 µg ABA test beverages also showed significant reductions in II by approximately –15% (100 mg FFE-50×, *P* = 0.046 and 600 mg FFE-10×, *P* = 0.022) compared to the glucose solution alone, but the reductions in GI did not reach statistical significance. There was a significant dose–response effect, with increasing dose of ABA producing a greater reduction in GI and II of the glucose reference drink (trend *P* < 0.01 for both GI and II). 

## 4. Discussion

The present study shows that the addition of FFE to a standard beverage significantly reduced postprandial glycemic and insulinemic responses compared to the glucose beverage alone. The higher doses of ABA (200 mg FFE-50× and 1200 mg FFE-10×) reduced glycemic and insulinemic responses by ~25% in healthy adults. Our results indicate that FFE in relatively small quantities (100–1200 mg) may be a useful food fortification or supplementation strategy to help reduce the GI of high-GI foods or diets. A recent systematic review and meta-analyses reported a 10 unit reduction in GI was sufficient to produce significant risk reductions in the development of type 2 diabetes [23]. Acute consumption of FFE with a food produced a clear dose-response effect with clinically relevant reductions in GI and II. 

Characterization of foods in terms of their GI and glycemic response has been a matter of continuous attention among the scientific community [24], as well as for individuals with insulin resistance. A number of different plant extracts have been shown to reduce postprandial glycemia or GI of common carbohydrates and carbohydrate-rich foods. Wang et al. reported that consumption of mulberry leaf extracts reduced the overall glycemic response, peak glucose concentrations at 30 min, and GI of glucose by 8.2% [25]. In our study, supplementation with FFE reduced GI to a greater extent (13%–25%) compared to mulberry leaf extracts. Moreover, the reduction in glycemia achieved with FFE extracts at low dosages is superior to several available functional fiber preparations that are required at higher dosages (≥5 g/day\) to achieve reduction in postprandial glycemia, which is only modest in many cases [26,27,28]. Soluble dietary fiber primarily acts by creating viscous gels that hinder glucose absorption, though some evidence exists for additional glucose-regulating effects in part through regulation of cholesterol and lipid levels in type 2 diabetes [29]. Soluble dietary fiber acts by creating viscous gels that hinder glucose absorption. However, addition of fiber can be associated with food palatability issues and often compromises the organoleptic appeal of foods. In addition, dietary fiber may increase the likelihood of gastrointestinal complaints, such as flatulence and diarrhea, due to its colonic fermentation properties [28]. In contrast, FFE has a different mechanism of action (ABA is a regulator of glucose disposal), does not create any viscosity issues or adverse gastrointestinal side effects, can be added to foods in relatively smaller quantities, and was well tolerated by all study participants.

Fruit and vegetable consumption patterns differ around the world, but we estimate ABA intake from these sources would range from approximately 160–260 µg ABA per day (on average fruits contain 0.62 µg ABA/g and vegetables contain 0.29 µg ABA/g [18]). The American Heart Association 2020 Strategic Goals recommend ≥4.5 servings/day of fruits and vegetables [30], which is estimated to provide ≥297 μg of ABA per day [18]. However, only 8% of the US adult population meets these dietary recommendations [31], suggesting that the majority of the population is consuming diets low in ABA, which could impact overall health outcomes. Dietary ABA has been shown to improve glucose tolerance in both experimental animals and healthy participants [9,19]. These published data in combination with our findings suggest that dietary ABA administration added into foods or beverages or potentially incorporated into the food matrix could be effective in improving glucose tolerance. The results of the present study show that FFEs containing ABA are an effective dietary intervention to produce significant reductions in GI and II of a high-GI glucose drink. Our data are comparable to results observed with synthetic ABA or a plant source of ABA [19]. Therefore, given the important role of low-GI diets in helping to improve glycemic control [32,33,34], consumption of FFE, such as ABALife™, could be a promising adjunctive dietary intervention for management of metabolic stress and chronic metabolic disorders.

The present study had certain limitations, including a small sample size of healthy participants with normal glucose tolerance and insulin sensitivity as well as a short duration, which only allowed the investigation of acute postprandial effects of the extracts. Randomized, placebo-controlled studies with larger numbers of participants and long-term trials are warranted to further confirm our observations. Specifically, studies including individuals with metabolic disorders, such as IGT, diabetes, or obesity, will help characterize the efficacy and engagement of LANCL2 by ABA in the target tissue. This further work may yield important new insights into preventive or therapeutic dietary strategies using ABA.

## 5. Conclusions

In summary, our study demonstrated that FFE standardized in ABA, when consumed in relatively small doses, can produce significant and clinically relevant reductions in postprandial glucose and insulin responses to a high-GI glucose drink. A similar dose-response reduction in GI and II values was observed for both the lower and higher extract concentrations, confirming that the FFE matrix did not influence the activity of ABA. Supplementation with FFE standardized in ABA, in the form of dietary supplements or functional beverages, may serve as a promising, novel nutritional intervention for the management of postprandial glucose homeostasis and metabolic disorders such as metabolic syndrome, prediabetes, and possibly diabetes and obesity.

## 6. Patents

A patent-pending process (EP17382616.5) was used to produce the dried fig fruit extracts (ABAlife^TM^, Euromed, Spain) investigated in this study.

## Figures and Tables

**Table 1:** Glycemic index (GI) and insulinemic index (II) values for the four test beverages containing ABALife™ fig fruit extracts relative to the reference glucose solution (GI and II = 100). Data are mean ± SEM, *n* = 10. The two lower doses of each fig fruit extract (FFE), 100 mg FFE-50× and 600 mg FFE-50×, contained 40 µg abscisic acid, and the two higher doses of each FFE, 200 mg FFE-50× and 1200 mg FFE-10×, contained 80 µg abscisic acid.

Test Food	GI Value	Mean GI Difference ^1^	II Value	Mean II Difference ^1^
Reference food (glucose drink)	100	⁻	100	⁻
ABALife^TM^ 50× (100 mg) + glucose drink	87 ± 6	−13	86 ± 5 ^#^	−14
ABALife^TM^ 50× (200 mg) + glucose drink	75 ± 6 *	−25	76 ± 6 *	−24
ABALife^TM^ 10× (600 mg) + glucose drink	86 ± 5	−14	84 ± 6 **	−16
ABALife^TM^ 10× (1200 mg) + glucose drink	77 ± 5 ^‡^	−24	76 ± 5 *	−24

^1^ Mean difference relative to the reference glucose drink. * *P* = 0.001 vs. reference drink; ^‡^ *P* = 0.002 vs. reference drink; ** *P* = 0.022 vs. reference drink; ^#^ *P* = 0.046 vs. reference drink.
